# Activity of Ivermectin and Its Metabolites against Asexual Blood Stage Plasmodium falciparum and Its Interactions with Antimalarial Drugs

**DOI:** 10.1128/aac.01730-22

**Published:** 2023-06-20

**Authors:** Achaporn Yipsirimetee, Phornpimon Tipthara, Borimas Hanboonkunupakarn, Rupam Tripura, Dysoley Lek, Krittikorn Kümpornsin, Marcus C. S. Lee, Jetsumon Sattabongkot, Arjen M. Dondorp, Nicholas J. White, Kevin C. Kobylinski, Joel Tarning, Kesinee Chotivanich

**Affiliations:** a Department of Clinical Tropical Medicine, Faculty of Tropical Medicine, Mahidol University, Bangkok, Thailand; b Mahidol Oxford Tropical Medicine Research Unit, Faculty of Tropical Medicine, Mahidol University, Bangkok, Thailand; c Centre for Tropical Medicine and Global Health, Nuffield Department of Medicine, University of Oxford, Oxford, United Kingdom; d National Center for Parasitology, Entomology and Malaria Control, Phnom Penh, Cambodia; e Wellcome Sanger Institute, Wellcome Genome Campus, Hinxton, United Kingdom; f Calibr, Division of the Scripps Research Institute, La Jolla, California, USA; g Mahidol Vivax Research Unit, Faculty of Tropical Medicine, Mahidol University, Bangkok, Thailand; h Department of Entomology, Armed Forces Research Institute of Medical Sciences, Bangkok, Thailand

**Keywords:** ivermectin, ivermectin metabolites, drug-drug interactions, antimalarial drugs, *Plasmodium falciparum*

## Abstract

Ivermectin is an endectocide used widely to treat a variety of internal and external parasites. Field trials of ivermectin mass drug administration for malaria transmission control have demonstrated a reduction of *Anopheles* mosquito survival and human malaria incidence. Ivermectin will mostly be deployed together with artemisinin-based combination therapies (ACT), the first-line treatment of falciparum malaria. It has not been well established if ivermectin has activity against asexual stage Plasmodium falciparum or if it interacts with the parasiticidal activity of other antimalarial drugs. This study evaluated antimalarial activity of ivermectin and its metabolites in artemisinin-sensitive and artemisinin-resistant P. falciparum isolates and assessed *in vitro* drug-drug interaction with artemisinins and its partner drugs. The concentration of ivermectin causing half of the maximum inhibitory activity (IC_50_) on parasite survival was 0.81 μM with no significant difference between artemisinin-sensitive and artemisinin-resistant isolates (*P* = 0.574). The ivermectin metabolites were 2-fold to 4-fold less active than the ivermectin parent compound (*P* < 0.001). Potential pharmacodynamic drug-drug interactions of ivermectin with artemisinins, ACT-partner drugs, and atovaquone were studied *in vitro* using mixture assays providing isobolograms and derived fractional inhibitory concentrations. There were no synergistic or antagonistic pharmacodynamic interactions when combining ivermectin and antimalarial drugs. In conclusion, ivermectin does not have clinically relevant activity against the asexual blood stages of P. falciparum. It also does not affect the *in vitro* antimalarial activity of artemisinins or ACT-partner drugs against asexual blood stages of P. falciparum.

## INTRODUCTION

In 2021, there were an estimated 247 million malaria cases and 619,000 malaria deaths worldwide according to WHO estimates (1). Six countries in the Greater Mekong subregion (GMS)—Cambodia, China (specifically Yunnan Province and the Guangxi Zhuang Autonomous Region), Laos, Myanmar, Thailand, and Vietnam—have all pledged to aim for malaria elimination by 2030. Malaria prevention and treatment in this region relies heavily on safe and effective antimalarial drugs. Artemisinin resistance in Plasmodium falciparum has emerged and spread widely throughout the GMS (2, 3). It has also recently emerged in Rwanda (4, 5) and Uganda (6–8). The spread of antimalarial drug resistance is one of the most important obstacles to malaria elimination. New drugs are needed urgently for malaria treatment and elimination.

Ivermectin is a well-established antiparasitic drug with endectocidal properties. The primary target of ivermectin is the glutamate-gated chloride (GluCl) ion channel in the muscle and nerves of invertebrates (9–11). Ivermectin binds with high affinity and prevents the closure of the GluCl channel leading to an influx of chloride with subsequent hyperpolarization of the cell, leading to flaccid paralysis and potential death. Ivermectin has been used widely in mass drug administration (MDA) for the eradication of onchocerciasis and lymphatic filariasis. Recently, ivermectin has been proposed for use in MDA for malaria transmission control as it has potent mosquito-lethal properties against *Anopheles* mosquitoes (12–16). In *Anopheles*, ivermectin at sublethal concentrations delays time to refeeding, decreases locomotor activity, reduces fecundity, and inhibits *Plasmodium* development in the vector which could further impact transmission (17–21). Using ivermectin as a complementary approach for vector control can target mosquitoes that have changed their feeding behavior or survive from conventional vector control measures (i.e., indoor residual spraying and long-lasting insecticidal nets). Ivermectin MDA field trials have shown promising results in reducing wild *Anopheles* survival (14, 15, 22) and human malaria incidence (22, 23).

Ivermectin significantly reduces survival of *Anopheles* malaria vector (21, 24). *In vitro* mosquito-killing activity of ivermectin was used to predict mosquito-lethal effect in humans by pharmacokinetic-pharmacodynamic modeling. The simulated time above the lethal concentration that kills 50% of mosquitoes (LC_50_) after single dose administration of ivermectin (400 μg/kg) was 0.4 and 1.1 days in Anopheles dirus and *Anopheles minimus* mosquitoes, respectively (21). However, a clinical trial in healthy Thai adults showed much greater mosquito-killing activity after single dose ivermectin (400 μg/kg) treatment compared to *in vitro* ivermectin-spiked blood (24). This suggests that uncharacterized ivermectin metabolites may possess mosquito-lethal effects (24). When evaluated using *in vitro* systems, ivermectin is primarily metabolized by cytochrome P450 3A4. More than 10 ivermectin metabolites, mostly hydroxylated and demethylated, were identified using human liver microsomes (25, 26). These metabolites; 3”-O-demethyl ivermectin (M1), 4-hydroxymethyl ivermectin (M3), and 3”-O-demethyl, 4-hydroxymethyl ivermectin (M6) were found in human blood (26).

Ivermectin affects *Plasmodium* development in the *Anopheles* vector. Laboratory studies demonstrated that ivermectin at sublethal concentration on *Anopheles* vector inhibits P. falciparum and Plasmodium vivax sporogony by reducing oocyst prevalence and intensity (19, 21, 27–29). It has been reported that ivermectin inhibits the liver stages of Plasmodium berghei, similar to the pre-erythrocytic effects of primaquine (30), and it was also shown to inhibit the development of liver schizonts and hypnozoites of Plasmodium cynomolgi when evaluated in *in vitro* models (31). Ivermectin impaired both the *in vitro* sexual and asexual blood stage development in P. falciparum (32–34). However, the effect of ivermectin against the asexual blood stage showed very different levels of response in P. falciparum K1 strain when using different assays; IC_50_ of 8 μg/mL (equivalent to 9.1 μM) using the [^3^H] hypoxanthine incorporation assay (33) and 0.32 μg/mL (equivalent to 0.37 μM) using P. falciparum Histidine-Rich Protein 2 (HRP2) enzyme-linked immunosorbent assay (32). It was proposed that ivermectin blocked nucleo-cytoplasmic shuttling of P. falciparum signal recognition particle components (34, 35). Asexual stage P. vivax maturation was impaired when incubated with ivermectin-treated human plasma at 4 h after 200 μg/kg ivermectin single dose administration (28).

Ivermectin has been combined with dihydroartemisinin-piperaquine in MDA campaigns against malaria in The Gambia (22). This intervention was shown to be safe, well tolerate, and effective in reducing the prevalence of malaria in the region. Ivermectin has also been combined with seasonal malaria chemoprevention in a cluster-randomized trial evaluating sulfadoxine-pyrimethamine plus amodiaquine in Burkina Faso and reported no safety concerns (23). Furthermore, a healthy volunteer trial reported a small increase in ivermectin exposure of approximately 25% when combined with dihydroartemisinin-piperaquine but with no effect of primaquine co-administration (24). The lack of substantial pharmacokinetic drug-drug interactions and safety signals are promising, but there is still a paucity on the information of possible pharmacodynamic drug-drug interactions. The current study investigated the effects of ivermectin and its metabolites against asexual blood stages of artemisinin-sensitive and artemisinin-resistant P. falciparum isolates and the pharmacodynamic interactions of ivermectin when combined with commonly used antimalarial drugs.

## RESULTS

Ivermectin parent compound, ivermectin aglycone, ivermectin monosaccharide, M1, M3, and M6 (Fig. S1) were selected to study antimalarial effects in this study. Antimalarial activity of ivermectin and its metabolites on asexual blood stage were investigated using a standard SYBR green I-based 72 h *in vitro* assay. Ivermectin parent compound showed antimalarial activity on asexual blood stage in a dose-dependent manner with mean (95% confidence interval) IC_50_ of 0.81 (0.67 to 0.95) μM and 0.81 (0.75 to 0.88) μM when tested against two artemisinin-sensitive and five artemisinin-resistant isolates, respectively (Table 1; Fig. S2). There was no significant difference in IC_50_ between artemisinin-sensitive and artemisinin-resistant isolates (*P* = 0.574). The IC_50_s of all ivermectin-related compounds, including its major metabolites, were 2-fold to 4-fold higher than ivermectin parent compound (Table 1). Ivermectin aglycone had the highest IC_50_ and all ivermectin metabolites showed less potency compared to ivermectin parent compound against all isolates (*P* < 0.001).

**TABLE 1:** Antimalarial effect of ivermectin and its metabolites on asexual blood stages of P. falciparum^*a*^

Parasite	*Pfk13* mutation	Ivermectin	Ivermectin aglycone	Ivermectin monosaccharide	3″-O-demethyl ivermectin (M1)	4-hydroxymethyl ivermectin (M3)	3″-O-demethyl, 4-hydroxymethyl ivermectin (M6)
Laboratory strain							
NF54	Wild type	0.63 (0.46 to 0.80)	2.55 (1.91 to 3.18)	1.88 (1.39 to 2.38)	1.99 (1.69 to 2.29)	1.68 (1.22 to 2.14)	2.57 (2.02 to 3.13)
Artemisinin-sensitive isolates							
ARN3G	Wild type	0.85 (0.55 to 1.10)	2.72 (2.56 to 2.87)	1.97 (1.56 to 2.39)	1.91 (1.78 to 2.05)	2.33 (1.78 to 2.87)	2.80 (2.42 to 3.18)
ARK1G	Wild type	0.76 (0.49 to 1.00)	2.79 (2.14 to 3.43)	2.14 (1.71 to 2.57)	2.02 (1.47 to 2.57)	1.82 (1.61 to 2.02)	2.35 (2.28 to 2.43)
Mean of all artemisinin-sensitive isolates (*n* = 2)		0.81 (0.67 to 0.95)	2.75 (2.57 to 2.93)	2.06 (1.87 to 2.24)	1.97 (1.80 to 2.13)	2.07 (1.74 to 2.41)	2.58 (2.30 to 2.86)
Artemisinin-resistant isolates							
APL4G	C580Y	0.74 (0.66 to 0.81)	3.02 (2.46 to 3.58)	2.01 (1.57 to 2.45)	2.03 (1.65 to 2.41)	1.98 (1.75 to 2.20)	2.66 (2.32 to 3.00)
APL5G	C580Y	0.76 (0.47 to 1.10)	4.23 (2.07 to 6.40)	2.97 (1.89 to 4.06)	1.91 (1.60 to 2.23)	2.20 (1.46 to 2.94)	2.85 (1.85 to 3.84)
APS2G	R539T	1.06 (0.87 to 1.20)	3.80 (3.53 to 4.06)	3.13 (3.04 to 3.23)	2.49 (2.04 to 2.94)	2.84 (2.60 to 3.08)	3.74 (3.24 to 4.24)
APS9G	C580Y	0.79 (0.65 to 0.92)	3.35 (2.60 to 4.10)	2.56 (2.30 to 2.82)	2.29 (1.89 to 2.69)	2.21 (1.98 to 2.44)	3.54 (3.05 to 4.03)
ARN2G	G449A	0.78 (0.63 to 0.92)	3.01 (2.25 to 3.78)	2.46 (1.81 to 3.11)	2.00 (1.89 to 2.10)	2.40 (1.96 to 2.84)	3.37 (2.81 to 3.93)
Mean of all artemisinin-resistant isolates (*n* = 5)		0.81 (0.75 to 0.88)	3.42 (3.10 to 3.74)	2.58 (2.34 to 2.82)	2.14 (2.00 to 2.28)	2.30 (2.14 to 2.47)	3.23 (2.97 to 3.49)

a All data are reported as mean IC_50_ (95% confidence interval), with the unit μM.

**FIG 1:**
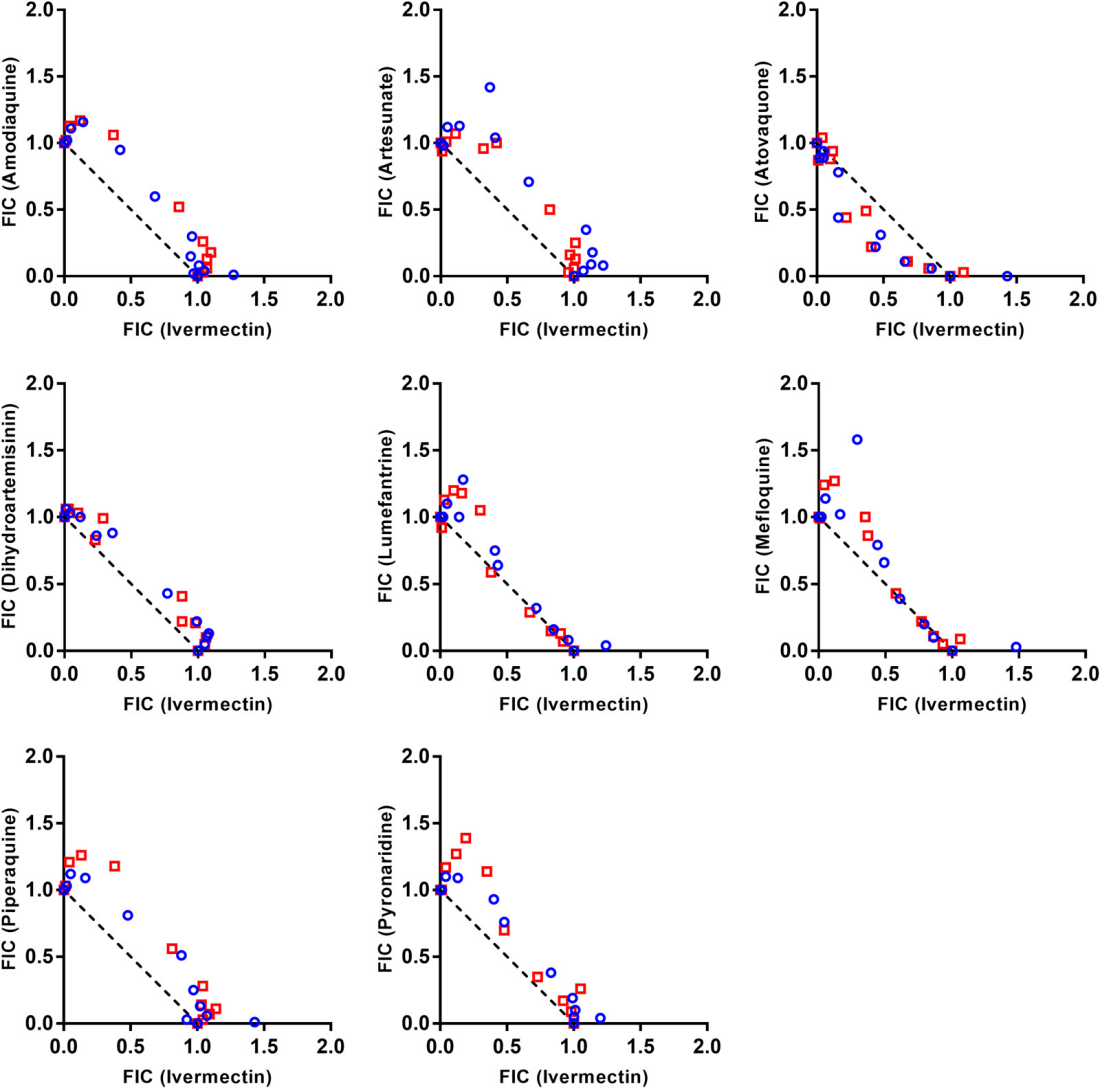
Pharmacodynamic interaction analysis. Isobologram analysis of ivermectin and antimalarial drugs against artemisinin-sensitive (blue circle symbol) and artemisinin-resistant (red square symbol) P. falciparum isolates, illustrated as fractional inhibitory concentration (FIC). The dashed line represents no interaction between the two drugs.

Ivermectin and antimalarial drug combination activity against P. falciparum was evaluated by a checkerboard analysis and presented as isobolograms and FIC indices. Eight combinations were tested with artemisinin-sensitive and artemisinin-resistant P. falciparum isolates. Isobologram analysis demonstrated no substantial interaction between ivermectin and amodiaquine, atovaquone, artesunate, dihydroartemisinin, lumefantrine, mefloquine, piperaquine, or pyronaridine (∑FIC > 0.5 and ≤4) (Fig. 1; Table 2).

**TABLE 2:** Antimalarial effects of ivermectin in combination with antimalarial drugs on asexual blood stages of P. falciparum^*a*^

Drug combination(Drug A – Drug B)	Artemisinin-sensitive isolates (*n* = 2; NF54, ARN3G)			Artemisinin-resistant isolates (*n* = 3; APL5G, APS2G, ARN2G)		
	IC_50_ of ivermectin, (x10^3^ nM)	IC_50_ of antimalarial, nM	∑FIC	IC_50_ of ivermectin, (x10^3^ nM)	IC_50_ of antimalarial, nM	∑FIC
Ivermectin – Amodiaquine	0.94(0.72 to 1.16)	11.42(7.94 to 14.90)	1.18(1.09 to 1.27)	1.18(0.82 to 1.53)	12.31(10.83 to 13.79)	1.21(1.13 to 1.30)
Ivermectin – Artesunate	0.93(0.77 to 1.10)	3.07(0.72 to 5.41)	1.31(1.18 to 1.44)	1.27(0.98 to 1.56)	3.90(2.24 to 5.57)	1.16(1.06 to 1.26)
Ivermectin – Atovaquone	0.79(0.70 to 0.88)	0.38(0.25 to 0.51)	0.89(0.73 to 1.06)	1.20(0.81 to 1.58)	0.62(0.21 to 1.04)	0.90(0.77 to 1.02)
Ivermectin – Dihydroartemisinin	1.07(0.83 to 1.32)	1.93(1.40 to 2.47)	1.15(1.10 to 1.19)	1.30(1.16 to 1.45)	1.92(1.77 to 2.06)	1.15(1.09 to 1.21)
Ivermectin – Lumefantrine	0.95(0.70 to 1.19)	7.40(2.11 to 12.70)	1.13(1.03 to 1.22)	1.41(0.99 to 1.83)	6.14(3.96 to 8.32)	1.09(0.98 to 1.21)
Ivermectin – Mefloquine	0.78(0.62 to 0.93)	10.96(4.05 to 17.87)	1.15(1.03 to 1.27)	1.13(0.92 to 1.27)	23.08(10.2 to 35.97)	1.14(1.02 to 1.25)
Ivermectin – Piperaquine	0.80(0.66 to 0.95)	12.94(10.09 to 15.78)	1.21(1.10 to 1.31)	1.06(0.84 to 1.27)	12.16(9.46 to 14.85)	1.26(1.15 to 1.37)
Ivermectin – Pyronaridine	0.95(0.83 to 1.07)	4.38(3.21 to 5.55)	1.18(1.11 to 1.25)	1.22(0.84 to 1.61)	2.74(1.88 to 3.59)	1.22(1.11 to 1.34)

a All data are reported as mean values (95% confidence interval).

The antimalarial activity of artesunate was further studied alone and in combination with a fixed dose of 50 ng/mL ivermectin by the trophozoite maturation assay. No difference was observed in IC_50_ values of artesunate alone or in combination with ivermectin in both artemisinin-sensitive (*P* = 0.385) and artemisinin-resistant (*P* = 0.546) isolates (Fig. S3).

## DISCUSSION

The World Health Organization (WHO) has recommended a range of interventions to achieve the elimination of malaria (36). Ivermectin has a significant mosquito-lethal effect on many species of *Anopheline* mosquitoes. (17–19, 21, 28, 37). It has been proposed that ivermectin be used as a complementary malaria vector control tool (38). Although several studies have reported that ivermectin has a clear concentration-dependent mosquito-lethal effect, resulting in a reduced incidence of malaria, no study has evaluated the pharmacodynamic drug-drug interactions of ivermectin and the commonly used antimalarial drugs against the asexual blood stage of *Plasmodium* parasites. The *in vitro* activity of ivermectin was assessed against P. falciparum laboratory strains and isolates. Previous reports on the effect of ivermectin on the asexual blood stage of P. falciparum have reported IC_50_ values ranging from approximately 0.021 μM to 9 μM (Table S1) (32–34, 37, 39). Differences in IC_50_ values between studies may be due to variations in parasite strains, drug exposure times, and methods of assessment. In this study, the antimalarial activity of ivermectin was evaluated against artemisinin-sensitive and artemisinin-resistant P. falciparum isolates; however, there was no correlation between the artemisinin resistance status of the isolate and the ivermectin IC50 (~0.8 μM). A study from Gabon P. falciparum isolates reported relatively low IC50 of 0.14 μM in a chloroquine-sensitive isolate JH26, and 0.021 μM and 0.13 μM in chloroquine-resistant isolates JH1 and JH13, respectively. Also, there was no correlation between the effect of ivermectin and chloroquine resistance (32).

Previous work identified ivermectin metabolites generated by liver microsomes, primary human hepatocyte, and human blood after ivermectin administration (26). In this current study, we examined how three primary ivermectin metabolites (M1, M3, M6), as well as ivermectin monosaccharide and aglycone, affected both artemisinin-sensitive and resistant parasites. Ivermectin was more potent than ivermectin aglycone, ivermectin monosaccharide, and all ivermectin *in vivo* metabolites (M1, M3, and M6). The effect of ivermectin aglycone, which lacks the sugar moiety and has a hydroxy-group at C-13 position, presented >90% parasite growth at 1 μM concentrations in this study. The reduction of effect due to these molecular modifications has been shown previously (37, 39).

A single dose of 5 mg/kg ivermectin had no effect on the blood stages of Plasmodium berghei in rodents, resulting in the same level of parasitemia, gametocytemia, and exflagellation as vehicle control (29). The impact of ivermectin on different *Plasmodium* developmental stages has been evaluated, and found to inhibit liver-stage development of P. berghei (30, 37, 39) and Plasmodium cynomolgi *in vitro* (31). Three doses of 10 mg/kg ivermectin inhibited approximately 80% of P. berghei liver infections and enhanced host survival in 80% of the treated mice (30). However, no causal prophylactic effect of ivermectin was observed for P. cynomolgi infections in macaques (0.3 to 1.2 mg/kg) (31) or P. falciparum infections in a controlled human malaria infection model of ivermectin administration at 400 μg/kg (40). Ivermectin inhibited the sporogony of P. falciparum (27) and P. vivax (19, 21) at sublethal concentrations to *Anopheles* vectors by reducing oocyst prevalence and intensity. It remains unclear if the drug acts on mosquito midgut physiology or interferes with sporogony development. Ivermectin and avermectin derivatives showed no activity against gametes. However, they exhibited inhibitory effects against the late sporogony process of ookinete and oocyst formation in a mosquito-free *in vitro* assay to study the direct drug effect on sporogony in Plasmodium berghei (29).

The optimal dosage and regimen of ivermectin are key parameters to maintain plasma ivermectin concentration at effective levels (41). A standard dose of ivermectin for onchocerciasis and lymphatic filariasis were 150 μg/kg and 200 μg/kg. In comparison, the tested doses of ivermectin trials for malaria transmission control varied from 150 to 600 μg/kg (13–16, 22, 23). Peak concentrations of 56.8 ng/mL ivermectin was observed after a single dose of 400 μg/kg in healthy Thai adults (24). While peak concentrations of 64.1 ng/mL and 105.2 ng/mL were reported in malaria patients after receiving a 3-day treatment of dihydroartemisinin-piperaquine and ivermectin at 300 and 600 μg/kg/day, respectively (42). At this dosage, plasma ivermectin showed potent mosquitocidal effects against *Anopheline* mosquitoes (24, 43). In contrast, the asexual blood stage IC_50_ observed in this study was approximately 0.8 μM (equal to 712 ng/mL), which is 6-fold to 12-fold higher than clinically relevant peak plasma ivermectin concentration at commonly used doses and regimens. Thus, it is unlikely that ivermectin MDA for malaria transmission control will impact asexual blood stage malaria parasites. In addition, ivermectin-treated parasites showed increasing trends of sexual commitment in a transgenic P. falciparum NF54 strain that expressed endogenous mScarlet-tagged AP2-G, a specific marker for sexually committed ring stages (44).

During the malaria transmission season, ivermectin MDA alone (14, 15) and in combination with artemether-lumefantrine (13) or dihydroartemisinin-piperaquine (16, 22, 24) or albendazole (15, 23) significantly reduced mosquito survival and malaria cases. Although ivermectin MDA was designed to target mosquitoes that feed on humans, it is important to address the interaction between ivermectin and antimalarial drugs on asexual blood stages of P. falciparum. For instance, antagonistic interactions have been observed *in vitro* on P. falciparum when combining ivermectin and doxycycline (45). The results presented here demonstrate that the parasite killing effects of the commonly used antimalarial drugs is not altered when combined with ivermectin when evaluated *in vitro*. This suggests that there is no clinically important pharmacodynamic drug-drug interactions to consider for possible MDA administrations. However, limited data are available on pharmacokinetic drug-drug interactions between ivermectin and antimalarial drugs. A healthy volunteer trial in Thailand showed relatively minor increases in the exposure to both piperaquine and ivermectin when co-administered, but there was no drug-drug interaction reported for primaquine (24). The combination of ivermectin and ACTs in MDA campaigns need further monitoring of drug efficacy and pharmacokinetic drug interactions in endemic area.

In conclusion, ivermectin and its metabolites showed no antimalarial effects at clinically relevant concentrations, although ivermectin demonstrated stronger antimalarial activity than its metabolites. Furthermore, neither artemisinin-sensitive nor artemisinin-resistant P. falciparum isolates exhibited pharmacodynamic interactions between ivermectin and commonly used antimalarial drugs. These findings support that ivermectin is unlikely to interfere with the antimalarial activity of the commonly used antimalarial drugs.

## MATERIALS AND METHODS

### Parasite culture.

Artemisinin-sensitive (*n* = 2) and artemisinin-resistant (*n* = 5) P. falciparum isolates were obtained from clinical studies conducted on the Thailand-Cambodia border (2, 46). All parasite isolates were mycoplasma free. Parasites were cultured at 5% parasitemia and 5% hematocrit in culture medium. Culture medium consisted of Roswell Park Memorial Institute (RPMI) 1640 medium (Sigma catalog no. R6504) containing 50 mg/L hypoxanthine (Sigma catalog no. H9377), 3 mg/L thiamine (Sigma catalog no. T1270), 6 mg/L L-ascorbic acid (Sigma catalog no. A5960), 30 mg/L CaCl_2_ (Sigma catalog no. C4901), 26 mg/L KH_2_PO_4_ (Merck catalog no. A681173), 16 mg/L MgSO_4_ (Sigma catalog no. M8150), 1 g/L d-glucose (Sigma catalog no. G7021), 5.96 g/L HEPES (Sigma catalog no. H3375), 2 g/L NaHCO_3_ (Sigma catalog no. S5761), and 10% human serum. Culture medium was replaced daily and incubated at 37°C in a 5% CO2 incubator.

### Ivermectin, ivermectin metabolites, and antimalarial drugs.

Ivermectin parent compound (Sigma, catalog no. I8898), ivermectin aglycone (Cayman Chemical, catalog no. 19442), ivermectin monosaccharide (Clearsynth, catalog no. CS-CM-00113) were purchased commercially. The ivermectin metabolite, 4-hydroxymethyl ivermectin (M3), was synthesized from parent compound (WuXi AppTec). The 3″-O-demethyl ivermectin (M1) structure could not be produced by chemical synthesis and therefore bacterial strains were used to generate the 3″-O-demethyl ivermectin (M1) from parent compound, and the 3″-O-demethyl, 4-hydroxymethyl ivermectin (M6) from M3 (Hypha Discovery). A previously developed LC-MS/MS method was used to confirm that the synthesized compounds contained the stated metabolites (26). Ivermectin compounds were prepared as stock solutions at 2 mg/mL in DMSO.

Artesunate (Artesunate for Injection; registration no. 1C 3/35 [N], Guilin No.2 Pharmaceutical Factory) was dissolved in 5% NaHCO_3_ at 60 mg/mL. Amodiaquine was dissolved in 70% ethanol at 1 mg/mL. Atovaquone and dihydroartemisinin were dissolved in DMSO at 1 mg/mL. Lumefantrine was dissolved in absolute ethanol at 1 mg/mL. Mefloquine and piperaquine were dissolved in 0.1 M H_3_PO_4_ at 1 mg/mL. Pyronaridine was dissolved in RPMI 1640 medium at 1 mg/mL. All antimalarial drugs were kindly provided by Worldwide Antimalarial Resistance Network (WWARN). Stock solutions were kept at −80°C and diluted with culture medium before the assay was set up.

### Drug sensitivity on asexual blood stage.

Ivermectin and ivermectin metabolites were prepared by a 2-fold serial dilution (0.02 to 10 μM) in RPMI 1640 medium supplemented with 0.5% AlbuMAX II (Thermo Fisher Scientific catalog no.11021045) in flat bottom 96-well plates at 50 μL/well. Asexual blood stage parasites, predominantly at the ring stage, were prepared at 1% parasitemia and 2% hematocrit. In each well, 50 μL of parasite suspension was added and gently mixed with the compounds. After 72 h of incubation, SYBR green I staining was used to detect parasite growth (47, 48). Each well was filled with 100 μL of 2×SYBR-green I lysis buffer (0.1% wt/vol saponin, Sigma catalog no.47036; 1%vol/vol Triton X-100, Bio-Rad catalog no. 161-0407; 5 mM EDTA, Sigma catalog no. E7889; and 20 mM Tris-HCl, Sigma catalog no. T5941). Plates were incubated in the dark for 30 min before the fluorescence signal was measured on a microplate reader (Synergy H1, BioTek) using a 485-nm excitation filter and a 520-nm emission filter. Assays were performed for at least three independent biological replicates with technical duplicates in each isolate. The percentage of parasite growth and IC_50_ were calculated using GraphPad Prism version 8. Statistical significance was determined by Student's *t* test and nonparametric Mann-Whitney U tests.

### Pharmacodynamic drug-drug interactions with antimalarial drugs.

The effects of ivermectin parent compound in combination with antimalarial drugs against the asexual blood stage of P. falciparum were evaluated using the checkerboard technique (49, 50). Briefly, two-dimensional checkerboard titration was prepared in flat-bottom 96-well plates in a volume of 50 μL. The assay plate was prepared with combinations of ivermectin (0.05 to 10 μM) and individual antimalarial drugs (0.20 to 100 nM amodiaquine, 0.10 to 50 nM artesunate, 0.02 to 10 nM atovaquone, 0.1 to 50 nM dihydroartemisinin, 0.39 to 200 nM lumefantrine, 0.78 to 400 nM mefloquine, 0.39 to 200 nM piperaquine, and 0.20 to 100 nM pyronaridine). Asexual blood stage parasites, predominantly at the ring stage, were prepared at 1% parasitemia and 2% hematocrit. In each well, 50 μL of parasite suspension was added to a final volume of 100 μL and gently mixed with the compounds. After 72 h of incubation, parasite growth was assessed by DNA content using a SYBR green I-based fluorescence staining (47, 48). Each well was filled with 100 μL of 2×SYBR-green I lysis buffer. Plates were incubated in the dark for 30 min before the fluorescence signal was measured on a microplate reader (Synergy H1, BioTek) using a 485-nm excitation filter and a 520-nm emission filter. The interactions between two compounds were evaluated using isobolograms and derived fractional inhibitory concentrations (FIC). The sum of FICs (ΣFIC) were calculated by a fraction of the IC50s in each drug combination and the IC50s of the single drug according to equation 1.

(1) $$\sum F\mathrm{IC}=\mathrm{FI}C_{A}\;+\;\mathrm{FI}C_{B}=\frac{IC_{50A+B}}{IC_{50A}}+\frac{IC_{50B+A}}{IC_{50B}}$$

where IC50_A+B_ is the IC50 of drug A in combination with drug B, IC50_B+A_ is the IC50 of drug B in combination with drug A, IC_50 A_ and IC_50 B_ are the IC_50_ of drug A and drug B alone, respectively. ΣFIC of ≤0.5, 0.5 < FIC ≤ 4 and FIC > 4 indicated synergistic, indifferent, and antagonistic effects, respectively (51, 52). The isobolograms and derived mean FIC values were calculated from at least three independent biological replicates with technical duplicates in each assay.

The antimalarial effect of artesunate when combined with a fixed dose of ivermectin was further investigated by the trophozoite maturation assay (53). Two-fold serial dilutions of artesunate alone (0.001 to 1 μM) and artesunate combined with a fixed dose ivermectin at 50 ng/mL, the observed clinical peak concentration of ivermectin after administration of ivermectin (150 μg/kg) (54), were prepared in flat-bottom 96-well plates. Parasites, predominantly at the ring stage, were prepared at 1% parasitemia and 2% hematocrit, and then incubated with the drug for 24 h. Thick and thin blood films were harvested and stained with Field’s stain. The staging of parasite development was investigated using a light microscope, and the numbers of trophozoites were counted per 100 parasitized red blood cells. Trophozoites were identified by morphology, size, nuclear/cytoplasm ratios, and visible pigment. IC_50_ was evaluated from the inhibition of the parasite development from ring to trophozoites compared to parasites in drug-free control wells using GraphPad Prism version 8. Statistical significance was determined by Student's *t* test and nonparametric Mann-Whitney U tests.
