# Supplementary material for: Activity of Ivermectin and Its Metabolites against Asexual Blood Stage Plasmodium falciparum and Its Interactions with Antimalarial Drugs
Source: Antimicrob Agents Chemother. 2023 Jun 20;67(7):e01730-22. doi: 10.1128/aac.01730-22 (PMC10368210; doi:10.1128/aac.01730-22)
Supplement: Supplemental file 1 — Supplemental material. Download aac.01730-22-s0001.docx, DOCX file, 0.2 MB [file aac.01730-22-s0001.docx]

# **Supplementary information**

## **Supplementary figure 1**

Structures of ivermectin and metabolites. Ivermectin is a mixture of at least 90% of 22,23-dihydroavermectin B1a and less than 10% of 22,23-dihydroavermectin B1b. Ivermectin B1a monosaccharide and ivermectin B1a aglycone was removed one and two sugar moieties from the structure. The demethylation of ivermectin B1a metabolite M1 occurred at C3’’ (highlighted in red) and the hydroxylation of ivermectin B1a metabolite M3 occurred at C4 (highlighted in red). Ivermectin B1a metabolites M6 structure was modified by demethylation at C3’’ and hydroxylation at C4 (highlighted in red).


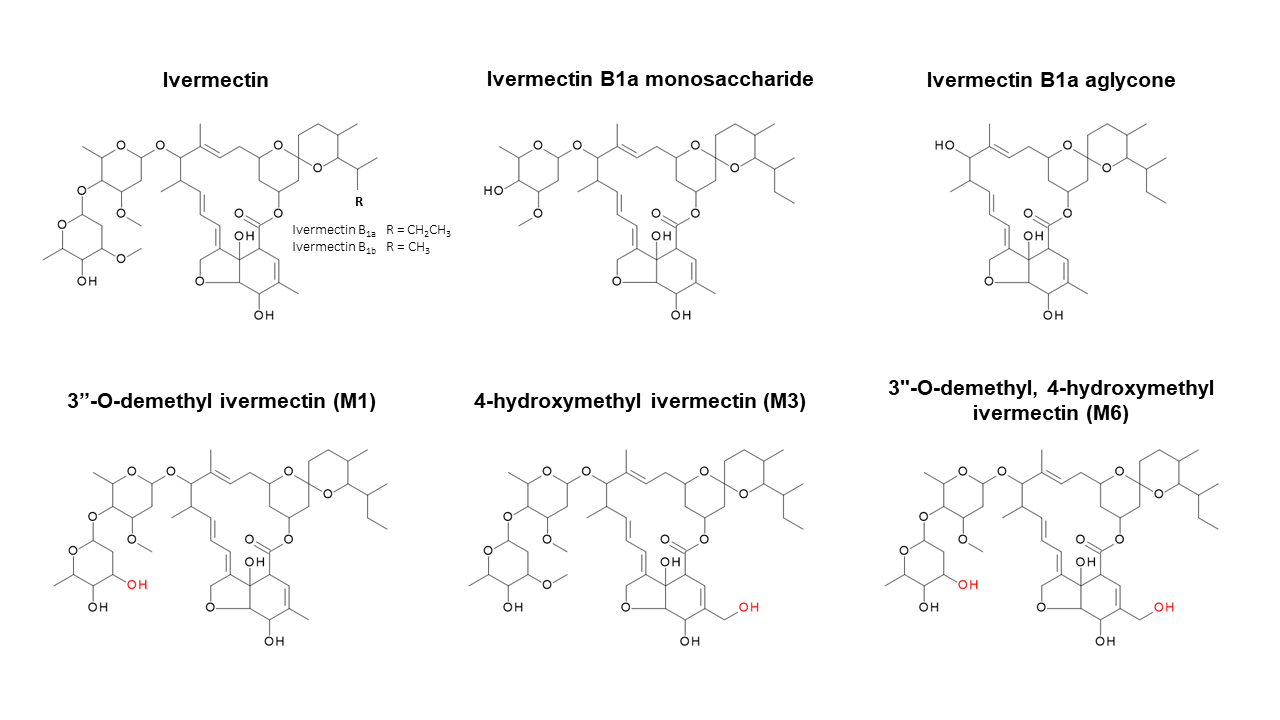


## **Supplementary figure 2**

Dose-response relationships (A) and forest plot of IC50 values (B) for ivermectin and metabolites against asexual blood stages of artemisinin-sensitive (n=2) and artemisinin-resistant (n=5) *P. falciparum.* The dashed line represents an ivermectin concentration of 56.18 nM which corresponds with peak plasma concentrations of ivermectin when administered at a single standard dose regimen of 150 µg/kg.

**(A)**

**(B)**


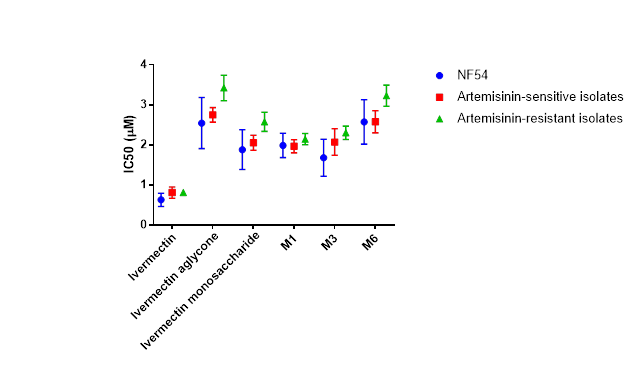


## **Supplementary figure 3**

Antimalarial activity of artesunate alone and in combination with a fixed dose of ivermectin at 50 ng/mL against artemisinin-sensitive and artemisinin-resistant *P. falciparum* isolates using trophozoite maturation assay. Mean (SD) IC50s of artesunate alone and in combination with ivermectin against artemisinin-sensitive isolates (N=2) were 2.17 (0.14) nM and 4.25 (2.56) nM, respectively. Mean (SD) IC50s of artesunate alone and in combination with ivermectin against artemisinin-resistant isolates were 5.65 (2.87) nM and 5.15 (3.35) nM, respectively. Bar and error bar represent the mean and SD of IC50 values.

## **Supplementary table 1**

*In vitro* effect of ivermectin against asexual blood stage of *P. falciparum*

| Parasite | IC50 | Method | Ref. |
| --- | --- | --- | --- |
| K1 | 8.0 µg/mL (equivalent to 9.1 µM) | [^3^H] hypoxanthine incorporation assay 48h drug incubation | 1 |
| Dd2  MCamp  3D7 | 2.85 µM  1.92 µM  1.56 µM | SYBR Green-1-based fluorescence assay 48h drug incubation | 2 |
| 3D7  Dd2  K1  JH1  JH13  JH26 | 100 nM  110 nM  365.3 nM  21.5 nM  126.4 nM  137.9 nM | Histidine-rich protein 2 (HRP2) enzyme linked immunosorbent assay 72h drug incubation | 3 |
| NF54 | 519.6 nM | Flow cytometric analysis using SYBR Green-I 48h drug incubation | 4 |
| NF54 | 359.6 nM | Flow cytometric analysis using SYBR Green-I 48h drug incubation | 5 |

**Reference**

1. Nasveld P, Russell B, Kotecka B, Rieckmann K. Lack of in vitro effect of ivermectin on Plasmodium falciparum. Southeast Asian J Trop Med Public Health. 2003;34(3):552-3.
2. Panchal M, Rawat K, Kumar G, Kibria KM, Singh S, Kalamuddin M, et al. Plasmodium falciparum signal recognition particle components and anti-parasitic effect of ivermectin in blocking nucleo-cytoplasmic shuttling of SRP. Cell Death Dis. 2014;5(1):e994.
3. de Carvalho LP, Sandri TL, José Tenório de Melo E, Fendel R, Kremsner PG, Mordmüller B, et al. Ivermectin Impairs the Development of Sexual and Asexual Stages of Plasmodium falciparum In Vitro. Antimicrob Agents Chemother. 2019;63(8).
4. Singh L, Fontinha D, Francisco D, Mendes AM, Prudêncio M, Singh K. Molecular Design and Synthesis of Ivermectin Hybrids Targeting Hepatic and Erythrocytic Stages of Plasmodium Parasites. Journal of Medicinal Chemistry. 2020;63(4):1750-62.
5. Singh L, Fontinha D, Francisco D, Prudêncio M, Singh K. Synthesis and antiplasmodial activity of regioisomers and epimers of second-generation dual acting ivermectin hybrids. Scientific Reports. 2022;12(1):564.
